# Automated Computer Vision and Dose-Response Modeling Improve Throughput and Accuracy of an *Ex Vivo* Functional Precision Medicine Platform

**DOI:** 10.21203/rs.3.rs-7303402/v1

**Published:** 2025-09-08

**Authors:** Noah Bell, Andrew Buckley, Breanna Mann, Xiaopei Zhang, Adebimpe Adefolaju, Rajaneekar Dasari, Rami Darwasheh, David E. Kram, Shawn Hingtgen, Andrew Satterlee

**Affiliations:** 1 Eshelman Innovation, University of North Carolina at Chapel Hill, Chapel Hill, NC, USA; 2 Eshelman School of Pharmacy, Division of Pharmacoengineering and Molecular Pharmaceutics, University of North Carolina at Chapel Hill, Chapel Hill, NC, USA; 3 Division of Pediatric Hematology-Oncology, University of North Carolina at Chapel Hill, Chapel Hill, NC, USA; 4 Corresponding Author and Lead Contact

**Keywords:** Computer vision, automation, machine learning, functional precision medicine, patient-derived

## Abstract

Functional Precision Medicine platforms, which investigate the dynamic behavior of a patient’s tumor *ex vivo* to inform personalized treatment, face unique obstacles to clinical translation. These include limited access to patient tissue and stringent demands for intra-platform accuracy and consistency. In this study, an automated data analysis pipeline addresses these concerns for an organotypic brain slice culture-based functional assay by combining computer vision and dose-response modeling approaches. A 99% reduction in analysis time increases the amount of patient tissue that can be processed on the platform. Comparing automated measurements to previously published manual results revealed that automation increased consistency both within experiments and across replicate experiments. This pipeline also explores implementing complex CV with limited resources, modeling a unique and diverse dataset, and validating automated analysis when no gold standard measurements exist, obstacles that hinder automation efforts across scientific disciplines.

## Graphical Abstract

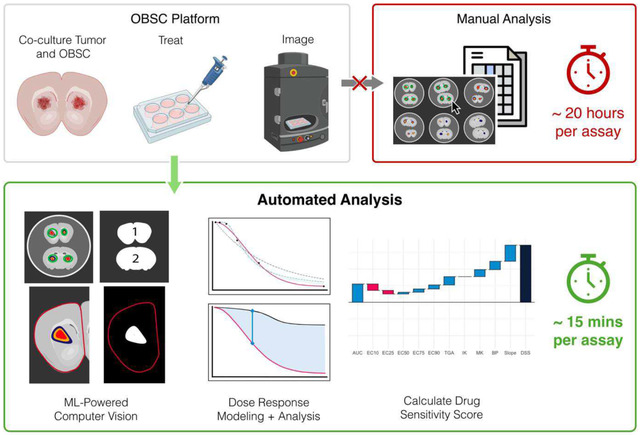

## Introduction

As biological and clinical research generates increasingly large and complex datasets, automation has become essential for efficient, consistent, and objective analysis. Advances in imaging technology have enabled the production of vast quantities of intricate imaging data; manual analysis of these images and downstream processing of the resultant data are often prohibitively time-consuming^1^. Moreover, manual image analysis is an inherently subjective process that often results in discrepancies among users^2,3^, and dim signals or nuanced features may be difficult to reliably detect by eye^4^. The increasing demand for efficient and objective analysis has spurred the development of numerous automated analysis tools. Some, like CellProfiler^5^ or ImageJ^6^, empower researchers across domains to design their own automated approaches, while others offer robust pipelines for domain-specific tasks^3,7,8,1^. However, if a unique platform lies in a gap unfilled by either general or specific automation solutions, the obstacle of manual analysis remains until a custom pipeline is designed. Researchers may encounter difficulties such as training an ML model^9,10^, validating their automated measurements when no gold standard dataset exists^11,12^, or modeling the resultant measurements^13,14.^

Our group encountered the bottleneck of manual analysis while developing an *ex vivo* platform for studying brain tumor treatment. This platform uses organotypic brain slice cultures (OBSCs) as living tissue substrates which host and sustain both tumor cell lines as well as living, uncultured brain tumor tissues resected from UNC Hospitals^15^. Engrafting patient tumors within the OBSCs’ natural brain architecture preserves the tumors’ original genetic and phenotypic characteristics and improves tumor survival *ex vivo*. In addition to studying genetic and phenotypic characteristics of engrafted tumors, we also treat each tumor with panels of therapeutics and directly assess antitumor potency and selectivity. We summarize both tumor response and off-target toxicity to the OBSCs themselves via a multiparametric Drug Sensitivity Score (DSS). Calculating DSSs for a single tumor sample against a panel of therapeutics required approximately twenty hours of active analysis, between (1) manually measuring a series of spatially dependent signals from various complex image types, (2) combining and processing the resultant measurements to generate dose-response data, and (3) calculating multiple parameters via linear interpolation from each set of dose-response curves to generate each DSS. This complexity significantly impacted our throughput; to continue moving the OBSC platform toward clinical validation, we needed an automated pipeline that maximized consistency and efficiency of analysis.

Here, we present a fully automated data analysis pipeline for an OBSC-based functional precision medicine platform, addressing many common obstacles to development of a customized pipeline. A user-friendly app ensures organized collection and storage of experimental metadata. A computer vision (CV) process, incorporating via API the machine learning (ML) web app Biodock^16,^ executes multimodal image analysis that is tailored to the various quantitative image-based data produced by the OBSC platform. Automated measurements of bioluminescence-based tumor kill were validated by comparison to previously published manual measurements^15^, with discrepancies further investigated to determine whether automation improved results. A multistep approach to Dose-Response Modeling (DRM) uses the resulting tumor and OBSC survival measurements to model treatment effect and extract relevant response parameters. Our data show that these models, along with improved statistical characterizations of dose-response, produce DSSs that more faithfully capture tumor-specific efficacy. Furthermore, DSS calculation for one tumor sample against a panel of therapeutics takes 15 minutes, rather than twenty hours. By increasing the efficiency and reliability of data analysis, this automated pipeline advances the translational potential of the OBSC functional diagnostic platform.

## Results

### Overview of the OBSC Assay

To measure therapeutic efficacy against tumor-OBSC co-cultures, micro-tumors are seeded on OBSCs and allowed to engraft for 24h before treatment is applied **(Supplemental Figure 1A).** If microtumors are generated from a cell line that already expresses the mCherry fluorescent marker, we take Day 1 Tumor images (D1-T) at this timepoint to establish an initial, pre-treatment size for each engrafted microtumor. If microtumors are generated from patient tumor tissues, these tumors are first rapidly infected with mCh/FLuc reporter genes (via lentiviral transduction which takes ~3 days to fully express) and thoroughly washed before engraftment onto OBSCs **(Supplemental Figure 1B)**. 24h after engraftment, microtumors are exposed to six increasing concentrations of a therapeutic, with survival calculated 72h after treatment initiation. Day 4 Tumor images (D4-T) are then taken to measure final microtumor survival by capturing the Fluc bioluminescent signal emitted by each surviving tumor cell.

In a parallel experiment, OBSCs without tumor are exposed to the same treatment and doses for the same amount of time, and Day 4 OBSC images (D4-O) are taken to measure treatment-induced toxicity using the fluorescent cell death marker Propidium Iodide **(Supplemental Figure 1C)**. The data from these three image types (D1-T, D4-T, and D4-O) are combined to calculate the percent survival of each microtumor or OBSC after treatment, measured as a percent of untreated tumor or OBSC survival.

On-target treatment-induced tumor kill is then compared to off-target toxicity to OBSCs across eleven dose-response parameters. These weighted parameters are combined into one Drug Sensitivity Score (DSS), where a score close to 100 indicates high tumor-specific killing and a score close to or below 0 indicates little or no tumor-specific killing.

### Metadata Input Form

Automation of data analysis in the OBSC pipeline required an organized way to store and manage experimental metadata, so that any results generated could be placed in their experimental context and easily accessed for future analysis. To ensure consistency and accuracy, we generated a simple application that allows users to generate correctly formatted metadata **(Supplemental Figure 2)**. Users can select tumor lines and treatments from an internal database that stores additional information about each tumor and treatment. After users define the experimental metadata and provide dose groups, the app creates a CSV file ready to be populated with data generated by the rest of the pipeline. For each microtumor engrafted on each OBSC, 45 fields of metadata are filled and stored in a remote database, and 31 fields are collected for each OBSC in an off-target toxicity assay. To-date, we have collected and stored 15,681 unique experimental results from 13,280 microtumors and 2,401 OBSCs in this database, totaling 672,031 individual data points. This manuscript details how analysis was automated for 886 microtumors and 1723 OBSCs previously published in *Cell Reports Medicine*^15^.

### Automating Image Analysis Using Machine Learning-Powered Computer Vision

**Figure 1.**
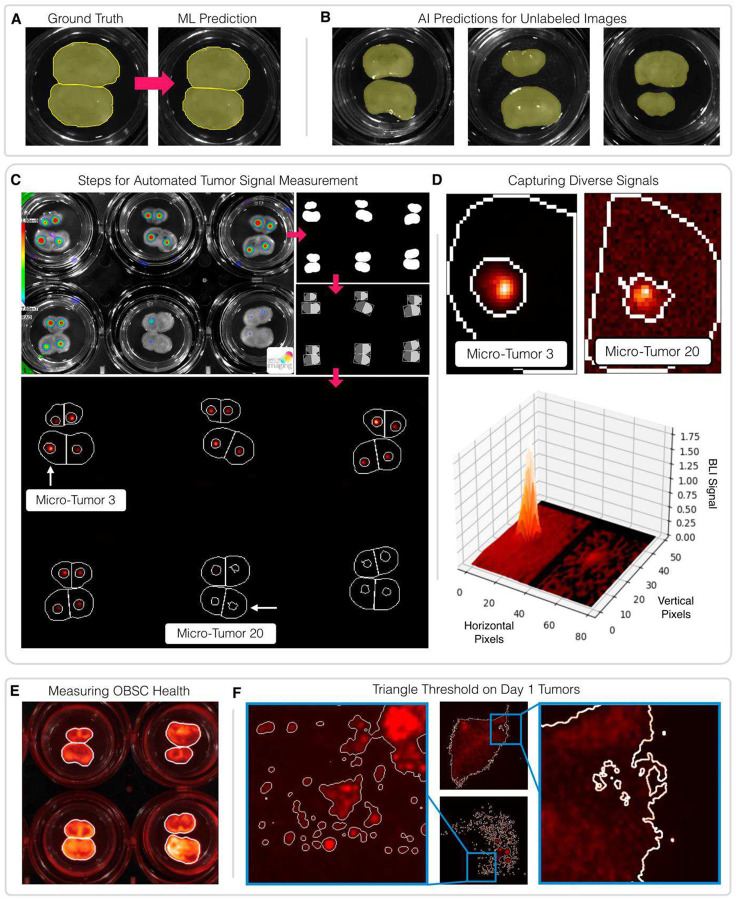
Visualization of Computer Vision Applied to Various Tumor Signals. (**A**) Two of the labeled OBSCs withheld from the ML training process in order to validate model performance. The ML model was able to precisely identify the two touching OBSCs in this validation image. (**B**) Three examples of the ML model creating OBSC masks for experiments analyzed in this paper. (**C**) A step-by-step representation of the D4T measurement process. OBSC masks are generated from the brightfield component of the D4T image, and these masks are bisected to distinguish between two hemispheres of the OBSCs. Thresholding is performed within each hemisphere to identify and measure the tumor present. (**D**) An example of how the process shown in (C) can identify a wide range of tumor sizes from the same image. The 3D surface plot highlights the 100-fold difference in signal magnitude between Micro-tumors 3 (left) and 20 (right). (**E**) OBSC masks generated by the same ML model can be used to measure Propidium Iodide fluorescence, a marker of cell death, across entire OBSCs. (**F**) Triangle thresholding on D1T images precisely distinguishes signal from background. The enhanced images feature adjustments to brightness and contrast to demonstrate how thresholding can detect features that might be obscured by computer display settings.

Of the three image types previously introduced, two (D4-T and D4-O) use the same format: a multimodal image of a six-well plate featuring a signal image, measuring a biologically relevant marker, overlaid on a grayscale image, which captures the visible OBSCs. A method to identify individual OBSCs in the grayscale image would facilitate further analysis for both image types. The grayscale images contained features that made conventional image analysis techniques difficult, such as bright areas of reflection off the plates and OBSCs that were occasionally touching the well walls or each other. Therefore, we decided to train an ML model to identify grayscale OBSCs on the web-based platform Biodock^16^. Biodock accelerates the development of ML models for biological applications by providing pre-trained models and an AI-assisted image labeling workflow. We trained an ML model on Biodock using 110 images containing 1,037 OBSCs, with 99 images used to train the model and 11 images reserved for model validation **(Supplemental Table 1).** All training and validation images were sourced from our own unpublished experiments to avoid overfitting our model to experimental images analyzed in this paper. This model achieved a Mean Average Precision over an IoU of 0.5–0.95 of 99.1 on the validation images **(Supplemental Table 2)**, reproducing the ground truth results with impressive accuracy (Figure 1A). This accurate identification of OBSCs in training images translated to unlabeled images from the experiments included in this paper (Figure 1B). Biodock’s extensive API functionality allowed us to incorporate the generated OBSC masks into custom, locally executed pipelines. The location of each mask in each image was used to determine which well, and therefore which dose group, each OBSC belonged to.

D4-T images feature two bioluminescent microtumors per OBSC, one on each hemisphere. To measure D4-T signals using the OBSC masks, we first bisected the masks, creating one mask per OBSC hemisphere. Then within each hemisphere mask we used the triangle thresholding algorithm^17,18^ to identify the tumor signal present (Figure 1C). This approach was able to successfully identify tumors with a 100-fold difference in signal magnitude within the same image (Figure 1D). Our approach also accounted for microtumors that had been completely killed, measuring the background noise in signal-free OBSC hemispheres. Biodock-generated OBSC masks were also used to calculate OBSC toxicity from D4-O image data. The average PI-derived fluorescence within each mask was measured to calculate dead cells within each OBSC (Figure 1E).

D1-T images were much simpler, each featuring an mCherry signal emitted by a single micro-tumor against a dark background. The triangle thresholding algorithm was again used to identify the mCherry signal. This algorithm was designed to differentiate dim signals from a large, dark background, and it performed well on D1-T images when tested against a variety of micro-tumor sizes and morphologies. Triangle thresholding even recognized tumor features that had been obscured by computer display settings (Figure 1F), demonstrating the ability of a relatively simple algorithm to identify signals more precisely and consistently than a human technician.

### Comparing Automated Image Analysis to Previously Published Manual Data

**Figure 2.**
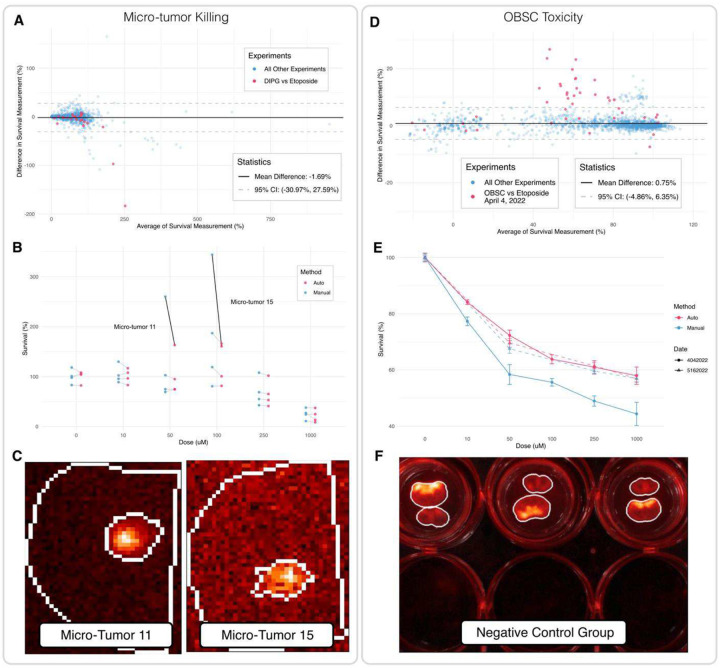
Comparing automated image analysis measurements to previous manual measurements. (**A**) A Bland-Altman graph comparing Manual and Automated Image Analysis results for each micro-tumor. The difference between Automated – Manual measurement is plotted on the y-axis, and the average between the two is plotted on the x-axis. The experiment highlighted in pink is DIPG vs. Etoposide 03/31/2022. (**B**) A Dose-Response plot for DIPG vs. Etoposide 03/31/2022 comparing the manual and automated results. Micro-tumors where Auto IA produced a large difference are highlighted. (**C**) The automatically generated masks for the highlighted micro-tumors above (11 on left, 15 on right). Micro-tumor 15 displays a low SNR, making an accurate ROI crucial. (**D**) A Bland-Altman graph comparing Manual and Automated Image Analysis results for each OBSC in the OBSC toxicity assays. The experiment highlighted in pink is OBSC vs. Etoposide 04/04/2022. OBSCs from positive control groups lie near 0% survival. (**E**) A Dose-Response graph showing how automation (pink) improves inter-experiment consistency between replicate experiments conducted in April (circles, solid line) and May (triangles, dashed line). (**F**) The automatically generated OBSC masks for the selected OBSC toxicity assay.

We next applied this image analysis pipeline to a set of experiments from a previously published paper^15^. This published dataset included tumor killing experiments performed on micro-tumors (from both established cell lines and patient tumor samples) co-cultured with OBSCs as well as the corresponding OBSC toxicity assays. We compared differences between the published, manually calculated survival of each micro-tumor and OBSC to our new automated measurements by generating Bland-Altman graphs (Figure 2A, 3D). Bland-Altman graphs were chosen for this data because, compared to simpler correlation calculations and plots, they offer a more accurate picture of how much two measurement methods disagree and whether the amount of disagreement is acceptable^19^. For each micro-tumor and OBSC, the *averag*e of the manual and automated measurements is plotted on the x-axis. The difference between the two measurements (Automated - Manual) is plotted on the y-axis. The mean difference is plotted as a black line, and the 95% confidence interval, conveying the distribution of differences, is plotted as dotted gray lines. The distinction between two measurements “agreeing” and “disagreeing” is arbitrary; in this study, we defined a difference of 10% survival as our agreement threshold. Measurements closer than 10% were considered to agree sufficiently, and measurements more than 10% different were investigated to determine whether automation had improved upon manual results or not.

Across 886 micro-tumors, the manual and automated survival measurements displayed a mean difference of −1.69% and a 95% Confidence Interval of (−30.9%, 27.59%) (Figure 2A). 723 of these measurements (82%) were within our 10% agreement threshold, while 163 measurements (18%) differed by more than 10%. In the experiment DIPG vs. Etoposide 3/31/2022 (Figure 2A, **pink)**, automation produced several different survival measurements, including one that was 183% lower than the previous manual measurement. A dose-response plot comparing Manual and Automated measurements for this experiment reveals that the automated measurements for micro-tumors 11 and 15 are more consistent with the survival of other replicate microtumors at those doses (Figure 2B): automation decreased the standard deviation from 90.1% to 41.9% in the 50 μM dose group and from 116.2% to 42.5% in the 100 μM dose group. Inspecting the automatically generated masks for these micro-tumors confirms that automation captured the signals precisely (Figure 2C). Four experiments, including DIPG vs. Etoposide, accounted for all disagreement greater than 50%, and in each of these cases the disagreement reflected a significant improvement in accuracy via automation **(Supplemental Figure 3A–D).** Comparisons of Manual and Automated measurements for all other microtumor experiments can be found in **Supplemental Figure 4.**

The manual and automated measurements of off-target OBSC toxicity show an even higher level of agreement, with a mean difference of 0.75% and a 95% confidence interval of (−4.86%, 6.35%) (Figure 2D). Here, 1,684 of 1,723 OBSCs (98%) had measurements that agreed within 10%; only 39 OBSCs (2%), all from two experiments, featured automated measurements that differed substantially from manual measurements. The experiment that showed the highest disagreement, with automated measurements as much as 26% higher than manual, was OBSC vs. Etoposide 04/04/2022 (Figure 2D, **pink)**. A second OBSC vs. Etoposide experiment was conducted on 05/16/2022, allowing us to investigate automation’s performance by measuring how automation impacted inter-experiment consistency (Figure 2E). The manual measurements for these two experiments (Figure 2E, **blue)** differed substantially at every shared dose group (50 μM: p = 0.051, 250 μM: p = 0.001, 1000 μM: p = 0.027), but automation led to remarkable consistency between the experiments (50 μM: p = 0.300, 250 μM: p = 0.851, 1000 μM: p = 0.751) (Figure 2E, **pink)**. The shift across all dose groups can be explained by shifts in the control group measurements. Since each dose group is normalized to the control group, it is crucial to obtain accurate control measurements. The automated masks for the negative control group in the 04/04/2022 experiment align with the fluorescent signals upon visual inspection (Figure 2F), producing additional confidence that the shift in results produced by automation is due to improved accuracy. The only other OBSC experiment with disagreements greater than 10% between manual and automation also featured improvements in the control group that impacted all dose groups **(Supplemental Figure 3E–F).**

### Multistep Approach to Dose-Response Modeling Allows for Robust Automation

After microtumor kill and off-target OBSC toxicity are measured, we estimate clinically relevant features of dose-response behavior (e.g., Effective Doses). Previously, we used linear interpolation between dose groups to estimate such parameters. However, linear interpolation only considers two dose groups at a time, leaving it vulnerable to local variations in data^20^.

**Figure 3.**
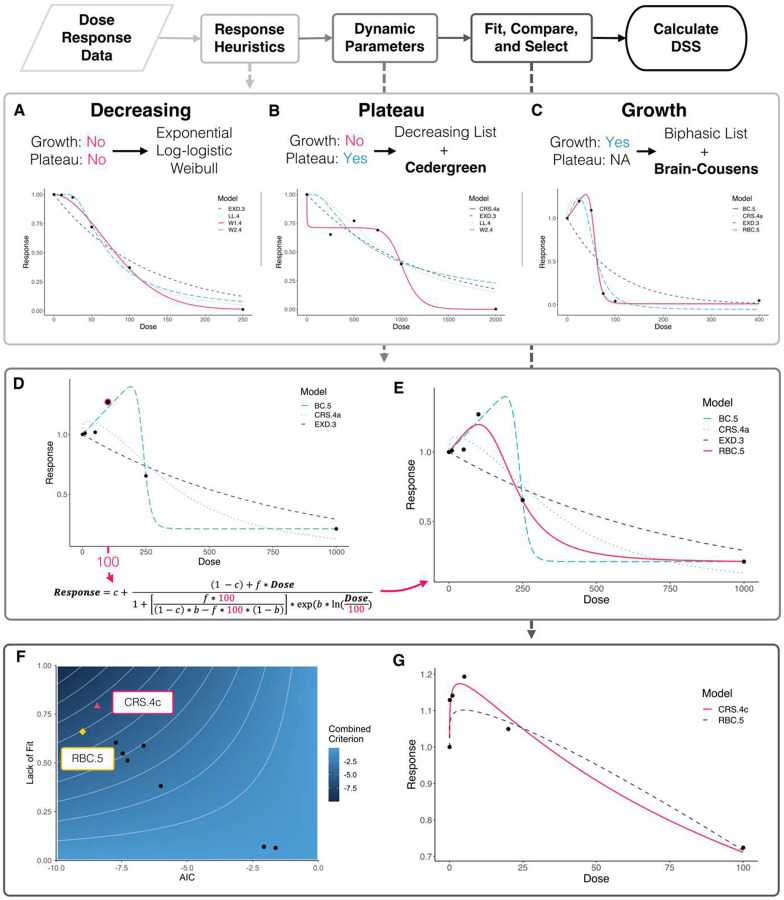
Automated Selection of Dose-Response Models. Through a combination of heuristics, rational parameter choices, and plausibility checks, the model fitting process could be controlled and customized for each data set. (**A**) Monotonic Decreasing data fit by a Weibull model (pink). (**B**) Biphasic data fit by a CRS model (pink). None of the standard decreasing models could accommodate all of the “plateau” points, and none reached the complete response seen at the highest dose. (**C**) Growth data displaying hormesis behavior fit by the standard Brain-Cousens model (pink). **(D)** Survival Data displaying hormesis behavior where standard models did not fit well to the data. To capture the hormesis behavior accurately, we found the dose of maximum growth and used it while fitting the Reparametrized Brain-Cousens model (RBC). (**E**) In this example, dynamically defining a parameter while fitting RBC led to a model that captured hormesis behavior without “overshooting” the observed peak survival. **(F)** This plot visualizes how we combined AIC and Lack-of-Fit to select the best fit model. A darker blue corresponds to a better Combined Criterion score. Each point corresponds to the scores of a given fitted model. In this example (U373KO vs. Trametinib), the chosen model, CRS.4c, had the best Combined score, even though it had a slightly lower AIC than the RBC model. (**G**) Qualitative confirmation that the selected CRS.4c model fits the underlying survival data better than RBC.5

Dose-response modeling (DRM), which fits a parametric model to an entire data set, provides a more biologically plausible and robust understanding of treatment efficacy^21,20.^ The open-source dose-response modeling package *drc*, written in R, features a wide variety of models and functions that enable rapid and accurate model fitting^22^. When using DRM instead of linear interpolation, the challenge is selecting an appropriate model for the data^20.^ Researchers often inspect a dataset and choose a model they expect to fit or choose a few models and compare their fit. We automated this process by writing a multi-step program that imitated an intuitive approach to DRM (Figure 3).

### Heuristic Categorization of Dose Response Behavior

Our microtumor and OBSC survival data displayed a variety of dose-response behaviors, some more complex than others. To ensure appropriate model fitting across all datasets, we heuristically sorted experiments into three categories, considering a different set of models for each category (Figure 3A–C, **Supplemental Figures 5–6).** In “**Decreasing”** experiments, higher doses led to increased tumor or OBSC kill until a final asymptote was reached (Figure 3A). Three families of simple models were tested on decreasing experiments. An experiment was placed in the **“Plateau”** category if a “plateau” separated two regions of killing (Figure 3B), where a plateau was defined as three or more dose groups where survival changed by less than 15% from dose to dose. In these experiments, the Cedergreen-Ritz-Streibig (CRS) model was considered in addition to the Monotonic Decreasing models^23.^ If the plateau effect was minor, a simpler decreasing model could still fit the data, thereby avoiding overfitting. “**Growth”** data exhibited more than 10% tumor growth at some dose (Figure 3C), and for these experiments we added the Brain-Cousens (BC) model to the CRS model and the list of decreasing models^24^.

### Defining Model Parameters Dynamically

Consistent automated model fitting required fine-tuning of parameter calculation. We defined a range of possible values for each parameter to ensure expected behavior, and we fixed certain parameters to specific values based our experimental constraints (e.g., survival at Dose = 0 was always 100%). We also defined certain parameter values *dynamically* based each experiment’s data. For example, the default parameterization of the BC and CRS models provide limited control of hormesis behavior (initial growth at low doses followed by killing at high doses), since no single parameter controls the size of the hormesis effect. Occasionally, these models failed to accurately capture the hormesis effect, especially in cases where only one or two doses featured growth (Figure 3D). To improve modeling of hormesis, we programmed a model which uses a Reparametrized version of BC (RBC)^25^. This model featured a parameter representing the dose where maximum growth occurred. Defining this parameter dynamically based on each experiment’s survival data helped RBC faithfully capture hormetic behavior while still fitting the subsequent tumor kill (Figure 3E).

A dynamic approach was also used to fit exponential decay models consistently. The model fitting process requires initial parameter guesses, and the quality of these guesses can determine whether a model fits successfully. By using a custom method based on linear interpolation **(Supplemental Figure 7)**, we could produce initial parameter guesses that resulted in consistent model fitting.

### Selecting the Final Model

After appropriate candidate models were chosen, we used a modified version of *drc*’s function *mselect* to fit and compare the models. *Mselect* calculates two statistics which we combined to select our final model. **Lack of Fit** measures how closely the given model aligns with the data points, and a value close to 1 is ideal^26^. **Akaike’s Information Criterion (AIC)** considers model complexity, favoring simpler models with fewer parameters^27^. Lower values of AIC represent more suitable models. In our dataset, we identified cases where AIC values were similar between two models but Lack-of-Fit indicated that one model aligned better with the underlying data. To account for this, we combined the two criteria (see Methods), allowing the more discriminatory measure to dominate (Figure 3F). This approach selected models that aligned with the underlying data while still considering model complexity (Figure 3G).

### Overall Impact of Automation on Drug Sensitivity Scores

Automatically fitted models were used to calculate dose-response parameters, which could then be combined into a single DSS for each therapeutic against each tumor. Automation also facilitated consistent, statistically driven methods for calculating dose-response parameters from the models and the underlying survival data. To determine the impact of automation on DSS calculation, we calculated automated DSSs for 31 previous tumor kill experiments, 20 conducted with tumor lines and 11 conducted with patient tumor tissues. We first evaluated the total change in DSS from the published manual process to the new automated pipeline, and then isolated the changes brought about by automating image analysis and dose response modeling respectively.

**Figure 4.**
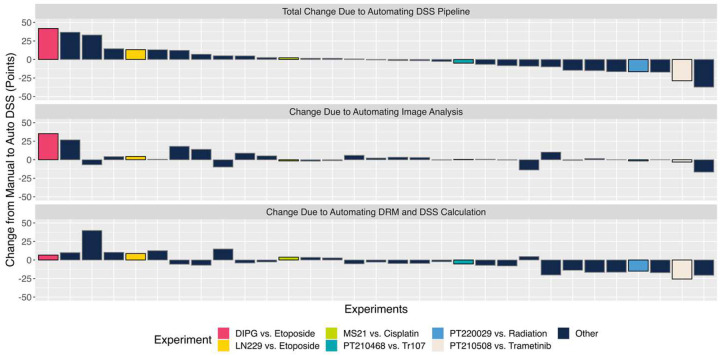
Automation’s Impact on DSS Scores. A bar graph demonstrating how automation changed DSS results across 31 OBSC platform experiments. All rows feature experiments in the same order, and all values represent the change from Manual to Automated. Six highlighted experiments will be explored further in Figure 6. **Top Row**: The change in DSS observed when Image Analysis is automated. **Middle Row:** The change in DSS observed when Automatic Dose-Response Models are fit to data and used to calculate DSS. **Bottom Row**: The total change in DSS from the completely manual calculations to the completely automated pipeline.

In 26 out of 31 experiments, automation produced a change in DSS smaller than 18 points (<10% of the total DSS range), with no clear bias in whether automation increased or decreased DSS scores (Mean Change = −0.03 points) (Figure 4, **top row**). Automating image analysis (Figure 4, **middle row)** only caused 2 experiments to shift by more than 18 points, while automating DRM and DSS calculation (Figure 4, **bottom row)** shifted 3 experiments by more than 18 points.

**Figure 5.**
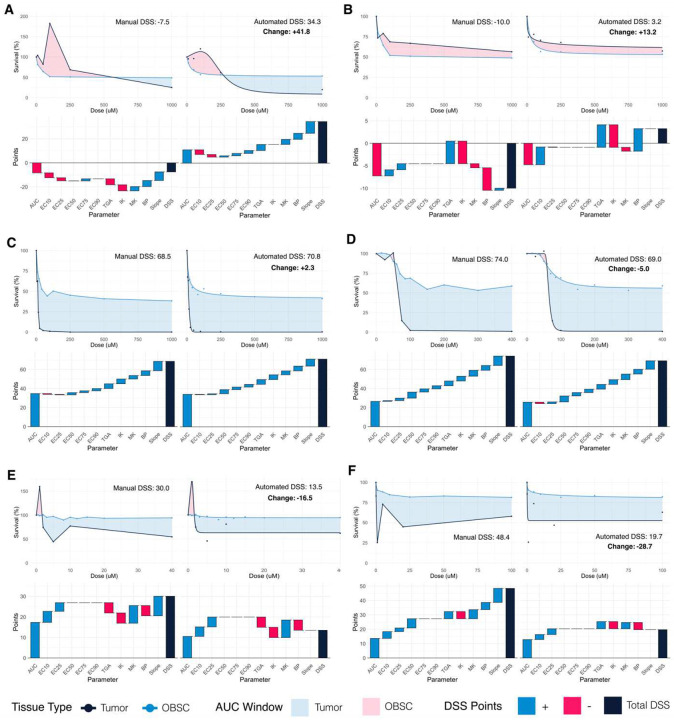
Automation’s Impact on Individual Experiments. An investigation of how automation impacted the DSS results in six experiments. Each experiment features four panels. **Top Left**: manual image analysis and linear interpolation. **Bottom Left**: a waterfall plot showing the manual DSS parameters (pink, light blue) and how they add up to the final DSS (navy). **Top Right:** Automated Image Analysis and Dose-Response Models. **Bottom Right:** A waterfall plot for the automated parameters and DSS. (**A**) DIPG vs. Etoposide, also featured in Figure 3. (**B**) LN229 vs. Etoposide (**C**) MS21 vs. Cisplatin (**D**) PT210468 vs. TR107 (**E**) PT220029 vs. Radiation (**F**) PT210508 vs. Trametinib

Six experiments have been highlighted to describe how automation impacts DSS calculation in greater detail (Figure 5, **larger images available in Supplemental Figures 8–13)**. In each panel, the previously published manual results are shown in the left column, and the new automated results are shown in the right; the top graphs show dose-response curves fit to the survival data, and the bottom waterfall plots visualize how the DSS is calculated from its component parameters. For DIPG vs. Etoposide (Figure 4, **pink)**, which was featured earlier in this paper (Figure 3A–C), automated image analysis measured less tumor growth at low doses, increasing the DSS by 41 points (Figure 5A). In the second highlighted experiment, LN229 vs. Etoposide, the original technician qualitatively decided that LN229 survival did not change substantially across the last three doses, so the tumor response was called biphasic (Figure 5B). However, the automated DSS process checks for a statistically significant difference between the last three groups. The 1000μM group was statistically significantly different from the previous two dose groups, so the response was determined to be *not* biphasic; this drove the 13-point increase in DSS. The next two highlighted experiments exemplify how automated DSSs matched the earlier manual results in many cases (Figure 5C,D). The experiment testing PT220029 vs. Radiation demonstrates how automated DRM and DSS calculation can affect the final DSS even when automated and manual image analysis matched (Figure 5E). Here, automated models yielded a smaller AUC window. The models also showed that, while there is some fluctuation in the underlying survival data, both the Tumor and OBSC response stalled at the highest doses, leading to no points being added for a difference in Slope. The final selected experiment evaluated Trametinib’s toxicity against PT210508, a patient-derived sample of Grade 2 Multiple Meningioma (Figure 4, **white)**. Here, the overall shift in DSS from 48.4 points (Manual) to 19.7 points (Automated) was driven by changes in how the Slope and Biphasic parameters are calculated (Figure 5F). The automated models also produced better estimates of Effective Doses, which further decreased the DSS.

## Discussion

Automation of image and data analysis meaningfully increased the translational potential of the OBSC functional diagnostic platform by improving accuracy, consistency, objectivity, and efficiency. Implementation of computer vision either maintained or improved the accuracy of measurements, as demonstrated by global observations and individual case studies. A multistep approach to Dose-Response Modeling, balancing between simplicity and detail, successfully fit models across a variety of response behaviors. The resultant Drug Sensitivity Scores, derived from these models and statistical tests on the raw data, more faithfully represented the relationship between treatment and tumor. Automation has decreased time spent on analysis from about 20 hours per assay to only 15 minutes. Our functional therapeutic efficacy data is stored in an organized database that facilitates future investigations into the clinical relevance of the OBSC platform.

Some analysis automation programs, like the computer vision software CellProfiler^5^, offer general modules that can be used for a wide variety of applications. Biodock fits in this category as well, making Machine Learning accessible for various biological imaging applications. Other programs fill a more specific niche, but one that is directly useful to a broader community of researchers. MyoSight^3^ analyzes cross-sections of muscle tissue; Neurite-J^7^ and Neurite Tracer^8^ both measure axonal growth in neurites. However, many assays – including our own - require a custom solution that accounts for the intricacies of the novel technology and application. While our pipeline is customized to our assay, the approaches used in this paper are generalizable to other applications. The integration of Biodock via API combined the capabilities of machine learning with the flexibility of executing custom scripts in a local Jupyter Notebook, making ML accessible to a broader range of scientists. Training our ML model on brightfield structures allowed us to apply our model in two separate pipelines and to account for large dynamic ranges and “missing” signals; these benefits could be realized by any researchers who use multimodal imaging systems like AMI or IVIS. Our approach of comparing automated and manual results and systematically investigating large differences could be adapted to validate other automation pipelines when no gold standard measurements exist.

Future developments to our pipeline will continue to streamline our workflow and improve our results. Our pipeline lacks a comprehensive User Interface (UI), but developing a UI will further enhance the efficiency and accessibility of automation. Automating DSS Calculation revealed that parameters which featured discrete categories (e.g. growth vs. no growth) sometimes led to unexpected DSS differences between similar-looking data. Making these parameters continuous would lead to more consistent and linear DSS calculations. When designing our model fitting approach, we aimed to faithfully represent tumor behavior while minimizing the risk of overfitting; however, we have not empirically confirmed that our approach is an ideal balance between the two. Measuring how a tumor responds to doses we have never tested before would reveal whether our approach accurately interpolates between dose groups. Finally, the relative clinical importance of each DSS parameter remains unclear. Translation of the DSS will require investigating the predictive power of various dose-response parameters in a clinical context.

Our automated analysis pipeline quickly generates accurate and consistent results. The time saved by automation frees researchers to process higher volumes of patient tissue, interpret results, and plan new experiments. It makes the OBSC platform well-poised for deep investigation into genetic and clinical correlations, maximizing our potential to uncover the utility of OBSC-tumor co-cultures as a clinically relevant model. Automation has thereby moved the OBSC assay toward our lab’s goal, and our field’s goal, of meaningfully shifting patient outcomes.

## Resource Availability

### Lead Contact

Further information and requests for resources and reagents should be directed to and will be fulfilled by the lead contact, Andrew Satterlee (satterle@e-mail.unc.edu).

### Materials Availability

This study did not generate new unique reagents.

### Data and Code Availability

Any information required to reanalyze the data reported in this paper and the code used to process the data is available on Mendeley Data DOI: 10.17632/s4mdscmh6w.1.

## Methods

### EXPERIMENTAL MODEL AND STUDY PARTICIPANT DETAILS

#### Secondary Analysis of Published Data

The current study is a secondary analysis of previously published data by our group^15^. Drug sensitivity data in the original paper were collected as described in the original publication^15^. All studies were approved by the necessary institutions including the Institutional Animal Care and Use Committee at the University of North Carolina-Chapel Hill and University of North Carolina’s Institutional Review Board. The study is reported in accordance with ARRIVE guidelines. Informed consent including relevant parental consent for minors was provided in compliance with governing rules and regulations.

### Method Details

#### Collecting Metadata

We created a User Interface (UI) for metadata input using the Shiny UI platform for R. We connected the Shiny app to locally stored CSV files which served as databases for tumor and treatment information. This information was used to populate drop-down lists that researchers could select from. Other fields, such as Dose groups, could be entered manually. Users could add up to nine dose groups. The number of rows in the resultant metadata CSV file corresponded to the number of micro-tumors or OBSCs in the experiment. Metadata that was usually consistent between experiments (e.g. thickness of OBSC, imaging equipment used, etc.) was automatically populated and hidden in the default form. However, users could choose to view and edit all metadata categories.

Each row in our database corresponds to one “target,” whether that be a micro-tumor in a tumor toxicity experiment or an OBSC in an OBSC toxicity experiment. Each row contained metadata that allowed the target to be placed in its experimental context, including the Treatment used, the Dose applied (including the units of concentration, e.g. μM or Gy), the date the experiment was started, the tumor type when applicable, etc. This allowed for experimental results to be easily queried later.

### Quantification and Statistical Analysis

#### Image Analysis

The image analysis pipeline was written in Python v3.10. The packages *NumPy* and *Pandas* were used extensively for storage and manipulation of data. *OpenCV* provided functions for image analysis. *Scikit-image* was used for image thresholding: 0ften, we needed to perform thresholding only on a specific segment of an image; we could pass the “masked” parts of the image directly to the threshold functions in *Scikit-image* without any further formatting of the data.

An Object-Oriented Programming (OOP) approach was used to orchestrate the various steps of image analysis. Different image types each had their own class, with custom measurement and data storage methods for each. An Experiment class was used to organize the various images present in an image. It also included methods that measured the various images present and handled Biodock API calls. OOP made development smooth, as custom functionality was built around the elements of the OBSC assay. Individual methods could be tweaked in class definitions without altering the overall performance of the pipeline.

We used Biodock to train an ML model to recognize OBSCs in grayscale images. Biodock guides users through multiple rounds of labeling and testing. Our final ML model was trained on 110 images containing 1037 OBSCs, all images being gathered from experiments that were not part of the data set used for validation in this paper. 99 images were used for model training, while 11 were reserved for model validation. The following data augmentations were applied: random horizontal flip, random vertical flip, random brightness, random contrast, random rotation, and random rescale. These augmentations improved the robustness of the resultant ML model when applied to new datasets. Contrast normalization was also applied. Images were uploaded to Biodock via their API protocol. API was also used to monitor the progress of new mask generation and to download mask results when they were ready. Biodock provides a UI for reviewing masks and editing and errors. Our pipeline allowed users to verify the results on Biodock and make any necessary edits before downloading the OBSC masks.

OBSC masks were sorted into separate wells based on the location of each mask. This accomplished by dividing the image into a grid, since the absolute locations of the wells were consistent from image to image. The top mask in each well was measured before the bottom mask.

For OBSC toxicity experiments, the OBSC masks were used to directly measure average fluorescence from each OBSC.

For Tumor toxicity experiments, the OBSC masks had to be split in two. This was accomplished by using a function in *OpenCV* that found rotated bounding boxes. A line was drawn down the center of these rotated boxes, dividing the OBSC mask into two hemispheres. Each hemisphere was analyzed separately by Triangle Thresholding, determining a threshold for each hemisphere that distinguished the tumor signal from the background.

#### Calculating Tumor and OBSC Survival

Raw image analysis results were loaded into R as data frames, which enabled calculations across large amounts of data. For tumor toxicity assays, percent survival was calculated by finding the average BLI signal from the negative control group (Dose = 0) and dividing every individual BLI result by that value. This means the negative control group had a defined mean survival of 100%, with other responses being measured relative to the negative control. OBSC toxicity assays include both a negative and positive control. The mean of the negative control group was subtracted from every individual fluorescent signal value, and then every signal was divided by the mean of the positive control group post-subtraction. This produced percent killing results, so subtracting this value from 100 yielded percent survival. An OBSC with a fluorescent signal equal to the mean of the negative control group would be interpreted as 100% survival, and an OBSC with a signal equal to the mean of the positive control would evaluate to 0% survival. A signal halfway in between the negative and positive means would become 50% survival.

#### Dose Response Modeling

Dose Response Models were fit and selected using the R package *drc*. Experiments were sorted into three categories by heuristically evaluating the mean responses. If any dose group had a mean survival equal to or greater than 110%, it was categorized as Growth. If there was no growth greater than 110%, the experiment could be Plateau or Decreasing. If an experiment had a plateau, where the mean survival did not change more than 15% between three or four dose groups but these dose groups were surrounded by changes >15%, it was categorized as Plateau. Any other experiments were categorized as Decreasing.

This categorization determined which models were considered when searching for a best-fit model. Each category had a corresponding list of models stored in a separate script. This allowed for definition of a fixed parameter list and other customizations (note: any parameters designated as NA were left unfixed and found by the model fitting function *drm*). There were accompanying lists containing parameter limits for each function, which bounded the range of possible values for non-fixed parameters (Inf or -Inf meant no limit was imposed). For Decreasing data, the following *drc* models were considered with the customizations described:

- LL.4
  - Parameters
    - b (Slope): (1, Inf)
      - Ensured that model would evaluate
    - c (Asymptote as Dose increases): (0, 1)
      - Ensured that model could not measure growth or predict <0% Survival
    - d (Asymptote at Dose = 0): fixed at 1
      - Defined survival = 100% at Dose = 0
    - e (Dose where inflection point occurred): (0.0001, Inf)
      - Ensured that model would evaluate
- W1.4
  - Parameters
    - b (Slope): (1, 50)
      - Prevented unreasonably steep slope and ensured that function behaved as expected
    - c (Asymptote as Dose increases): (0, 1)
      - Ensured that model could not measure growth or predict <0% Survival
    - d (Asymptote at Dose = 0): fixed at 1
      - Defined survival = 100% at Dose = 0
    - e (Dose where inflection point occurred): (0.0001, Inf)
      - Ensured that model would evaluate
- W2.4
  - Parameters
    - b (Slope): (−50, −0.3)
      - Prevented unreasonably steep slope and ensured that function behaved as expected. Note that the slope values must be negative for this version of the Weibull function.
    - c (Asymptote as Dose increases): (0, 1)
      - Ensured that model could not measure growth or predict <0% Survival
    - d (Asymptote at Dose = 0): fixed at 1
      - Defined survival = 100% at Dose = 0
    - e (Dose where inflection point occurred): (0.0001, 10000)
      - Ensured that model would evaluate
- EXD.3
  - Parameters
    - c (Asymptote as Dose increases): (0, Inf)
      - Ensured that model could not predict <0% Survival, but allowed for modeling exponential growth
    - d (Asymptote at Dose = 0): fixed at 1
      - Defined survival = 100% at Dose = 0
    - e (Steepness of decay): (0.0001, 10000)
      - Ensured that model would evaluate
  - Custom self-starter function, as described in Results

Plateau list added these versions of the CRS function to the functions considered for Decreasing (the variations a, b, and c correspond to fixed alpha parameters of 1, 0.5, and 0.25 respectively):

- CRS.4a, CRS.4b, CRS.4c (lower limit set at 0)
  - Parameters
    - b (steepness of post-hormesis decay): (1, 250)
      - Prevented unreasonably steep slopes
    - d (Asymptote at Dose = 0): (0.95, 1.05)
      - Couldn’t fix any CRS parameters, so this kept the Survival close to 100% at Dose = 0
    - e (controls “overall” size of model; larger values just make the function look bigger, but the overall shape stays the same): (0.000001, Inf)
      - Ensured that model evaluated
    - f (height of hormesis peak): (−Inf, Inf)
      - Allowing negative numbers makes capture of Two-Stage behavior possible
- CRS.5a, CRS.5b (free lower limit)
  - Parameters
  - b (steepness of post-hormesis decay): (1, 250)
    - Prevented unreasonably steep slopes
  - c (Asymptote as Dose grows larger): (0, 10)
  - d (Asymptote at Dose = 0): (0.95, 1.05)
    - Couldn’t fix any CRS parameters, so this kept the Survival close to 100% at Dose = 0
  - e (controls “overall” size of model; larger values just make the function look bigger, but the overall shape stays the same): (0.000001, Inf)
  - f (height of hormesis peak): (−10, Inf)

The Hormesis list added the following functions:

- BC.5
  - Parameters
    - b (Slope): (1, Inf)
    - c (Asymptote as Dose increases): (0, Inf)
    - d (Asymptote at Dose = 0): fixed at 1
    - e (controls “overall” size of model; larger values just make the function look bigger, but the overall shape stays the same): (0, Inf)
    - f (Height of Hormesis peak): (0.000001, Inf)
- RBC.5 (reparametrized version of BC.5711)
  - Parameters
    - b (Slope): (1, 50)
    - c (Asymptote as Dose increases): (0, Inf)
    - d (Asymptote at Dose = 0): fixed at 1
    - M (Dose at which hormesis peak occurs): defined dynamically based on data
    - f (Height of Hormesis peak): (0.000001, Inf)
  - Equation
    - $\text{Response}=c+(1-c)+f*\text{Dose}1+\left[f*M(1-c)*b-f*M*(1-b)\right]*\text{exp}(b*\text{In}(M100)\text{Response}=\text{c}+1-\text{c}+\text{f}*\text{Dose}1+\text{f}*\text{M}1-\text{c}*\text{b}-\text{f}*\text{M}*1-\text{b}*\text{exp}(\text{b}*\text{In}(M100)$

Once the appropriate model list was chosen, the candidate models were compared using the *drc* function *mselect*. This function had to be slightly modified to account for the pre-defined fixed parameters, parameter limits, and self-starting functions. *Mselect* produces multiple measures of model fit, including AIC and Lack-of-fit. AIC takes model complexity into account to avoid overfitting, with more negative values corresponding to better fit. Lack-of-fit (LoF) measures the statistical alignment of the model to all data points (considering replicate values) without considering model complexity. LoF is interpreted like R^2^, where a value closer to 1 signifies good fit. We ranked models according to a “combined criterion,” where lower values signified a better fit:

- if AIC was positive, Combined Criterion = AIC/LoF
  - Lower LoF leads to a larger CC value, higher LoF leads to smaller CC
- If AIC was negative, Combined Criterion = AIC * LoF
  - Higher LoF leads to a more negative CC, while lower LoF leads to a less negative CC

Using this piecewise function ensures that a more negative AIC and a LoF closer to 1 are always rewarded. Combining the values like this allowed us to choose models that fit the data better when AIC values were comparable, and it allowed us to pick models with better AICs when LoF values were comparable.

After ranking the fitted models by the combined criterion, we tested the models, starting with the best fit, to make sure that they didn’t overshoot or undershoot the range of observed data. We eliminated a model if it predicted a survival higher than 1.1 * the highest mean survival in the observed data. We also eliminated a model if it predicted a survival lower than the lowest mean survival - 50% Survival. The model with the lowest CC value that also passed these tests was chosen as the model of best fit.

Chosen models were plotted using the R package *ggplot*. The models were saved as .Rdata objects so that they could be loaded for future analysis.

#### DSS Calculation

The DSS is calculated as a weighted sum of various “windows” which compare the response of a tumor and the OBSC to a given treatment. Each window took a value between −1 (indicating greater treatment effect on the OBSC) and 1 (indicating greater treatment effect on the tumor). Each of the eleven parameters is described below:

- **EDxx Window**: The gap between the tumor and OBSC Survival values at the dose where xx% killing is observed in the tumor. The EDxx of the tumor was found numerically by finding where the fitted model predicted xx% killing. Windows were found for ED10, ED25, ED50, ED75, and ED90. OBSC survival was then evaluated at this dose. The window was found using the following piecewise function:
  - If OBSC health > 100% at EDxx:
    - EDxx Window = 1 (max value)
  - If OBSC health > Tumor Health at EDxx:
    - EDxx Window = (Tumor Kill - OBSC Kill)/Tumor Kill
  - If Tumor health > OBSC Health at EDxx:
    - EDxx Window = (Tumor Kill - OBSC Kill)/OBSC Kill
- **AUC Window**: The difference between the AUC of the Tumor model and the OBSC model. The AUC of each was found by numerically integrating the fitted models. AUC Window was calculated using the following piecewise function:
  - If OBSC AUC > Tumor AUC (corresponding to more tumor kill):
    - AUC Window = (OBSC AUC - Tumor AUC)/OBSC AUC
  - If OBSC AUC <= Tumor AUC:
    - AUC Window = (OBSC AUC - Tumor AUC)/Tumor AUC
- **Tumor Growth Acceleration (TGA):** A test for whether treatment accelerated tumor growth at any point.
  - If Max Survival > 150%:
    - TGA Window = −1
  - Else, If Max Survival > 125%:
    - TGA Window = 0
  - Else:
    - TGA Window = 1
- **Incomplete Kill (IK):** A test for whether treatment achieved complete tumor kill
  - If Survival at Highest Dose > 25%:
    - IK Window = −1
  - Else, if Survival at Highest Dose > 10%:
    - IK Window = 0
  - Else:
    - IK Window = 1
- **Max Kill (MK):** Compares Tumor Kill at the highest dose (TK) to OBSC Kill at Highest Dose (OK)
  - If TK > OK: MK Window =
    - (TK - OK)/TK
  - If OK > TK: MK Window =
    - (TK - OK)/OK
- **Biphasic (BP):** Checks for whether the highest three doses are statistically significantly different from each other.
  - If significantly different:
    - BP Window = 1
  - If not significantly different:
    - BP Window = −1
- **Slope:** Compares slope of tumor model to slope of OBSC model. If the tumor model reaches 50% kill, then the slope is evaluated at the Tumor’s ED50. Otherwise, the slopes are compared at the highest dose. A greater slope magnitude indicates higher treatment efficacy, so the values below represent the absolute value of the slope (slopes are always negative)
  - If Tumor Slope > OBSC Slope:
    - Slope Window = (Tumor Slope - OBSC Slope)/Tumor Slope
  - If Tumor Slope < OBSC Slope:
    - Slope Window = (Tumor Slope - OBSC Slope)/OBSC Slope

These window values are multiplied by weights which control the relative contribution of each window. The window weights are:

- AUC: 35
- MK, IK, and ED50: 10
- BP and all other EDxx: 5

This means the greatest possible DSS is 100, and the lowest possible value is −100. All constituent windows are recorded along with the final DSS.

## Supplementary Files

This is a list of supplementary files associated with this preprint. Click to download.

- BelletalSupplementalTables.pdf
- SupplementalFigures.docx
- BelletalSupplementaryMethods.docx

## References

[1] Park C. F. Automated neuron tracking inside moving and deforming C. elegans using deep learning and targeted augmentation. Nat. Methods 21, 142–149 (2024). 38052988 10.1038/s41592-023-02096-3 PMC13082739

[2] Heye T. Reproducibility of dynamic contrast-enhanced MR imaging. Part II. Comparison of intra- and interobserver variability with manual region of interest placement versus semiautomatic lesion segmentation and histogram analysis. Radiology 266, 812–821 (2013). 23220891 10.1148/radiol.12120255

[3] Babcock L. W., Hanna A. D., Agha N. H. & Hamilton S. L. MyoSight-semi-automated image analysis of skeletal muscle cross sections. Skelet. Muscle 10, 33 (2020). 33198807 10.1186/s13395-020-00250-5 PMC7667765

[4] Nath T. Using DeepLabCut for 3D markerless pose estimation across species and behaviors. Nat. Protoc. 14, 2152–2176 (2019). 31227823 10.1038/s41596-019-0176-0

[5] Carpenter A. E. CellProfiler: image analysis software for identifying and quantifying cell phenotypes. Genome Biol. 7, R100 (2006). 17076895 10.1186/gb-2006-7-10-r100 PMC1794559

[6] Rueden C. T. ImageJ2: ImageJ for the next generation of scientific image data. BMC Bioinformatics 18, 529 (2017). 29187165 10.1186/s12859-017-1934-z PMC5708080

[7] Torres-Espín A., Santos D., González-Pérez F., del Valle J. & Navarro X. Neurite-J: an image-J plug-in for axonal growth analysis in organotypic cultures. J. Neurosci. Methods 236, 26–39 (2014). 25124852 10.1016/j.jneumeth.2014.08.005

[8] Pool M., Thiemann J., Bar-Or A. & Fournier A. E. NeuriteTracer: a novel ImageJ plugin for automated quantification of neurite outgrowth. J. Neurosci. Methods 168, 134–139 (2008). 17936365 10.1016/j.jneumeth.2007.08.029

[9] Barbierato E. & Gatti A. The challenges of machine learning: A critical review. Electronics 13, 416 (2024).

[10] Tufail S., Riggs H., Tariq M. & Sarwat A. I. Advancements and challenges in machine learning: A comprehensive review of models, libraries, applications, and algorithms. Electronics 12, 1789 (2023).

[11] Myhre P. L. External validation of a deep learning algorithm for automated echocardiographic strain measurements. Eur. Heart J. Digit. Health 5, 60–68 (2024). 38264705 10.1093/ehjdh/ztad072 PMC10802824

[12] Giannoulatou E., Park S.-H., Humphreys D. T. & Ho J. W. K. Verification and validation of bioinformatics software without a gold standard: a case study of BWA and Bowtie. BMC Bioinformatics 15 Suppl 16, S15 (2014). 10.1186/1471-2105-15-S16-S15 PMC4290646 25521810

[13] Burgoon L. D. & Zacharewski T. R. Automated quantitative dose-response modeling and point of departure determination for large toxicogenomic and high-throughput screening data sets. Toxicol. Sci. 104, 412–418 (2008). 18441342 10.1093/toxsci/kfn083

[14] Tansey W. Dose-response modeling in high-throughput cancer drug screenings: an end-to-end approach. Biostatistics 23, 643–665 (2022). 33417699 10.1093/biostatistics/kxaa047 PMC9007438

[15] Mann B. A living ex vivo platform for functional, personalized brain cancer diagnosis. Cell Rep. Med. 4, 101042 (2023). 37192626 10.1016/j.xcrm.2023.101042 PMC10313921

[16] AI Software Platform. Biodock. (Biodock, 2024).

[17] Zack G. W., Rogers W. E. & Latt S. A. Automatic measurement of sister chromatid exchange frequency. J. Histochem. Cytochem. 25, 741–753 (1977). 70454 10.1177/25.7.70454

[18] Bankhead P. Analyzing Fluorescence microscopy images with ImageJ. (2014).

[19] Bland J. M. & Altman D. G. Statistical methods for assessing agreement between two methods of clinical measurement. Lancet 1, 307–310 (1986). 2868172

[20] Kappenberg F. Guidance for statistical design and analysis of toxicological dose-response experiments, based on a comprehensive literature review. Arch. Toxicol. 97, 2741–2761 (2023). 37572131 10.1007/s00204-023-03561-w PMC10474994

[21] Ritz C., Baty F., Streibig J. C. & Gerhard D. Dose-response analysis using R. PLoS ONE 10, e0146021 (2015). 26717316 10.1371/journal.pone.0146021 PMC4696819

[22] Ritz C. & Streibig J. C. Bioassay Analysis using R. J. Stat. Softw. 12, (2005).

[23] Cedergreen N., Ritz C. & Streibig J. C. Improved empirical models describing hormesis. Environ. Toxicol. Chem. 24, 3166–3172 (2005). 16445100 10.1897/05-014r.1

[24] Brain P. & Cousens R. An equation to describe dose responses where there is stimulation of growth at low doses. Weed Res. 29, 93–96 (1989).

[25] Abbaraju V. D., Robinson T. L. & Weiser B. P. Modeling Biphasic, Non-Sigmoidal Dose-Response Relationships: Comparison of Brain-Cousens and Cedergreen Models for a Biochemical Dataset. arXiv (2023) doi:10.48550/arxiv.2308.08618.

[26] Nonlinear regression analysis and its applications. (John Wiley & Sons, Inc., 1988). doi:10.1002/9780470316757.

[27] Cavanaugh J. E. & Neath A. A. The Akaike information criterion: Background, derivation, properties, application, interpretation, and refinements. WIREs Comp Stat 11, e1460 (2019).

